# Heterokont Predator *Develorapax marinus* gen. et sp. nov. – A Model of the Ochrophyte Ancestor

**DOI:** 10.3389/fmicb.2016.01194

**Published:** 2016-08-03

**Authors:** Vladimir V. Aleoshin, Alexander P. Mylnikov, Gulnara S. Mirzaeva, Kirill V. Mikhailov, Sergey A. Karpov

**Affiliations:** ^1^Belozersky Institute for Physico-Chemical Biology, Lomonosov Moscow State UniversityMoscow, Russia; ^2^Kharkevich Institute for Information Transmission Problems, Russian Academy of SciencesMoscow, Russia; ^3^Institute of Animal Physiology, Biochemistry and NutritionKaluga, Russia; ^4^Institute for the Biology of Inland Waters, Russian Academy of SciencesBorok, Russia; ^5^Institute of Gene Pool of Plants and Animals, Uzbek Academy of SciencesTashkent, Uzbekistan; ^6^National University of UzbekistanTashkent, Uzbekistan; ^7^Zoological Institute, Russian Academy of SciencesSt. Petersburg, Russia; ^8^St. Petersburg State UniversitySt. Petersburg, Russia

**Keywords:** *Develorapax marinus*, ochrophyte ancestor, molecular phylogeny, ultrastructure, prey size

## Abstract

Heterotrophic lineages of Heterokonta (or stramenopiles), in contrast to a single monophyletic group of autotrophs, Ochrophyta, form several clades that independently branch off the heterokont stem lineage. The nearest neighbors of Ochrophyta in the phylogenetic tree appear to be almost exclusively bacterivorous, whereas the hypothesis of plastid acquisition by the ancestors of the ochrophyte lineage suggests an ability to engulf eukaryotic alga. In line with this hypothesis, the heterotrophic predator at the base of the ochrophyte lineage may be regarded as a model for the ochrophyte ancestor. Here, we present a new genus and species of marine free-living heterotrophic heterokont *Develorapax marinus*, which falls into an isolated heterokont cluster, along with the marine flagellate *Developayella elegans*, and is able to engulf eukaryotic cells. Together with environmental sequences *D. marinus* and *D. elegans* form a class-level clade Developea nom. nov. represented by species adapted to different environmental conditions and with a wide geographical distribution. The position of Developea among Heterokonta in large-scale phylogenetic tree is discussed. We propose that members of the Developea clade represent a model for transition from bacterivory to a predatory feeding mode by selection for larger prey. Presumably, such transition in the grazing strategy is possible in the presence of bacterial biofilms or aggregates expected in eutrophic environment, and has likely occurred in the ochrophyte ancestor.

## Introduction

Heterokonta is one of the major groups of eukaryotes and encompasses organisms with a wide variety of life styles – from autotrophic diatom algae and kelps to heterotrophic bicosoecid flagellates and parasitic oomycetes. Autotrophic heterokonts form a bulk of the group’s described species, and are presumed to comprise a single monophyletic cluster Ochrophyta. By contrast, heterotrophic lineages of heterokonts do not form a single group, and are instead found as several clades that branch off the heterokont stem lineage preceding the divergence of ochrophytes (Leipe et al., 1994, 1996; Riisberg et al., 2009; Yubuki et al., 2010, 2015). The evolution of heterokonts, where autotrophs occupy the tree “crown” and heterotrophs form a grade of early-diverging lineages, implies either multiple independent transitions to heterotrophy from the ancestral autotrophic lifestyle assumed by the “Chromalveolata” hypothesis (Cavalier-Smith, 1999) or an event of plastid acquisition in the heterotrophic ancestors of the Ochrophyta. The question of autotrophic versus heterotrophic ancestry of heterokont lineages remains open (Mukhina, 2014; Petersen et al., 2014; Stiller et al., 2014; Archibald, 2015, for recent review). One of the difficulties with the latter evolutionary scenario is the apparent prevalence of bacterivory in close relatives of ochrophytes, which precludes the prerequisite for plastid acquisition – the ability to engulf eukaryotic prey. It was shown experimentally that there are optimum prey size spectra for various species of predatory protists (Hansen, 1992; Jakobsen and Hansen, 1997; Montagnes et al., 2008; Nielsen and Kiørboe, 2015). Among recent heterotrophic and mixotrophic species there are those that are able to consume both bacterial and eukaryotic prey (Strom, 1991; Burkholder et al., 2008; Piwosz and Pernthaler, 2010; Zhang et al., 2013; Lee et al., 2014; Mitra et al., 2016) while others are limited by the size of prey cells, with some eukaryotes lacking the ability to graze on bacteria (Jonsson, 1986; Hansen and Calado, 1999; Montagnes et al., 2008). Bacterivorous flagellates have two alternative feeding strategies: whereas some species are grazing on single bacterial cells only, other species are capable of ingesting aggregates of bacterial cells (Sibbald and Albright, 1988). Flagellates, adhering to the second strategy, are able to consume bacterial biofilms, including those that grow on solid substrates (Böhme et al., 2009; Erken et al., 2012).

Here, we present a new genus and species of marine free-living heterotrophic heterokont *Develorapax marinus* – an obligatory predator feeding on kinetoplastids and to lesser extent by ingesting bacterial cells. Its 18S and 28S rRNA genes are clustered in the phylogenetic analyses with those of *Developayella elegans* (Tong, 1995). The bacterivorous marine flagellate *Developayella* together with few environmental sequences forms a clade with unstable position in the phylogenetic trees (Beakes et al., 2012): it is either grouped with the oomycete/hyphochytrid clade (Leipe et al., 1996; Cavalier-Smith and Chao, 2006; Riisberg et al., 2009; Yubuki et al., 2010), or is nested between the ochrophytes and the oomycete/hyphochytrid clade (Yubuki et al., 2015). Thus, on the one hand, free-living *Developayella*-like organisms might contain ancestral characters common with parasitic oomycetes (Beakes et al., 2012) or with a larger part of heterokonts (Moriya et al., 2000). On the other hand, *Developayella*-like organisms might possess features shared by the ancestors of the ochrophyte lineage. We suggest that the transition from the bacterivorous to predatory mode of feeding behavior in the *Developayella/Develorapax* clade might have paved the way for the development of endosymbiosis with a rhodophyte cell (Bhattacharya and Medlin, 1995; Delwiche et al., 1995; Daugbjerg and Andersen, 1997; Keeling, 2004) in the initially bacterivorous ancestors of ochrophytes.

## Results

**FIGURE 1:**
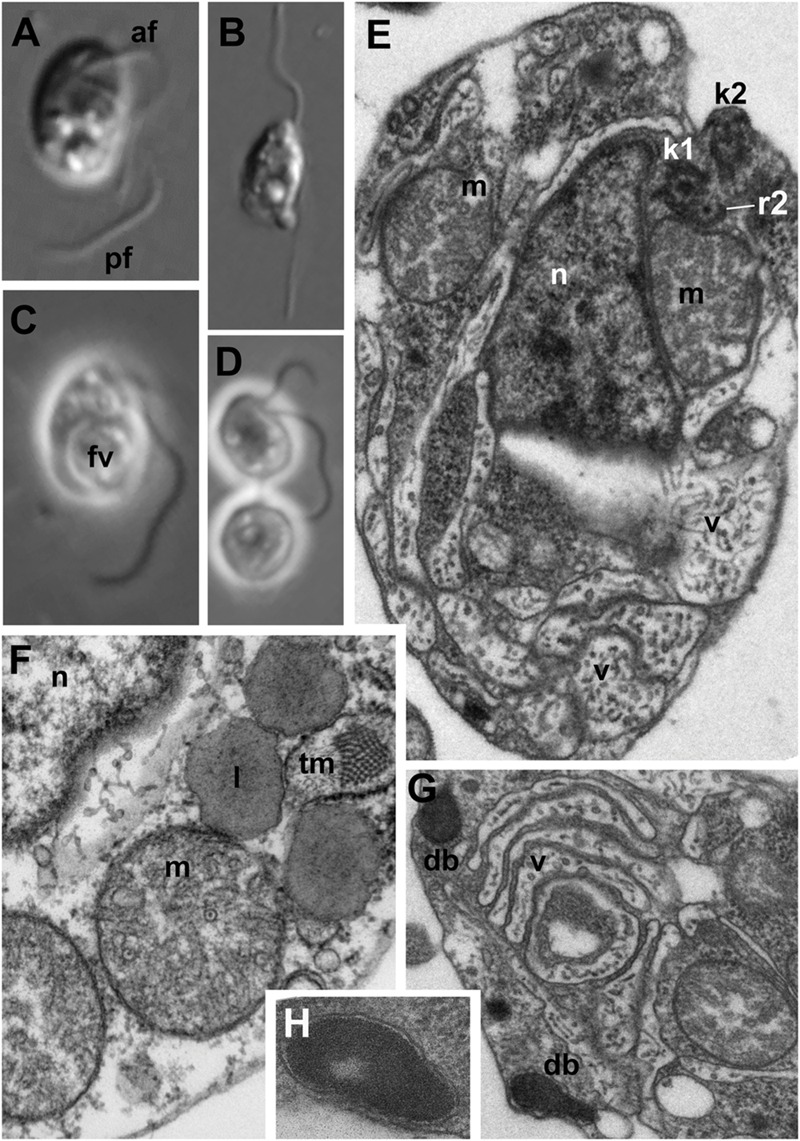
**Light **(A–D)** and electron **(E–H)** microscopic images of *Develorapax marinus*. (A,C)** Trophont, **(B)** swimming cell, **(D)** two daughter cells still connected by thin cytoplasmic bridge (arrowhead). **(A,B)** DIC, **(C,D)** phase contrast. **(E)** General view at longitudinal section, **(F)** some organelles and portion of nucleus at higher magnification (arrow shows an axial filament in the crista), **(G)** structure of vacuoles and dense bodies, **(H)** dense body at higher magnification. Scale bar: **(A–D)** 5 μm; **(E,G)** 500 nm; **(F)** 300 nm; **(H)** 125 nm. Abbreviations: af, anterior flagellum; b, bacteria; db, dense bodies; fv, food vacuole; ga, Golgi apparatus; kp, kinetoplast of the prey; k1, kinetosome of posterior flagellum; k2, kinetosome of anterior flagellum; l, lipid globule; m, mitochondrion; mp, mitochondrion of the prey; n, nucleus; nu, nucleolus; pf, posterior flagellum; pr, prey; ps, pseudopodium; r1–r4, microtubular flagellar roots; smt, secondary microtubules of r3; tm, tubular mastigonemes; tp, transversal plate; v, vacuole.

General morphology of *Develorapax marinus* (see description in taxonomy section; **Figure 1**) is similar to that of *Developayella elegans*, the only other described and studied member of this clade (Tong, 1995). The result of molecular phylogenetic analysis with small and large subunit rRNA gene sequences confirms that *Develorapax marinus* and *Developayella elegans* are sisters. Therefore, we describe the light and electron microscopic observations of *D. marinus* by comparing it with *D. elegans. D. marinus* differs from *D. elegans* in the details of morphology and biological peculiarities, which justifies its description as a new genus and species.

### Light Microscopy

Cells of *D. marinus* are oval in shape, and measure 7–10 μm in length (vs. 3.5–8.5 μm for *D. elegans*) and 4–6 μm in width (vs. 2–6 μm for *D. elegans*). Two flagella are inserted at the deepest part of a pronounced depression on the right-hand side of the anterior half of the cell (**Figure 1**), similar to *Developayella*. The anterior flagellum is 1.5 of the cell length, and the posterior flagellum is about twice the cell length. Similar to *D. elegans*, the posterior flagellum is fastened along the bottom of the depression, whilst the anterior flagellum emerges freely, and nucleus is visible close to the point of flagella insertion.

**FIGURE 2:**
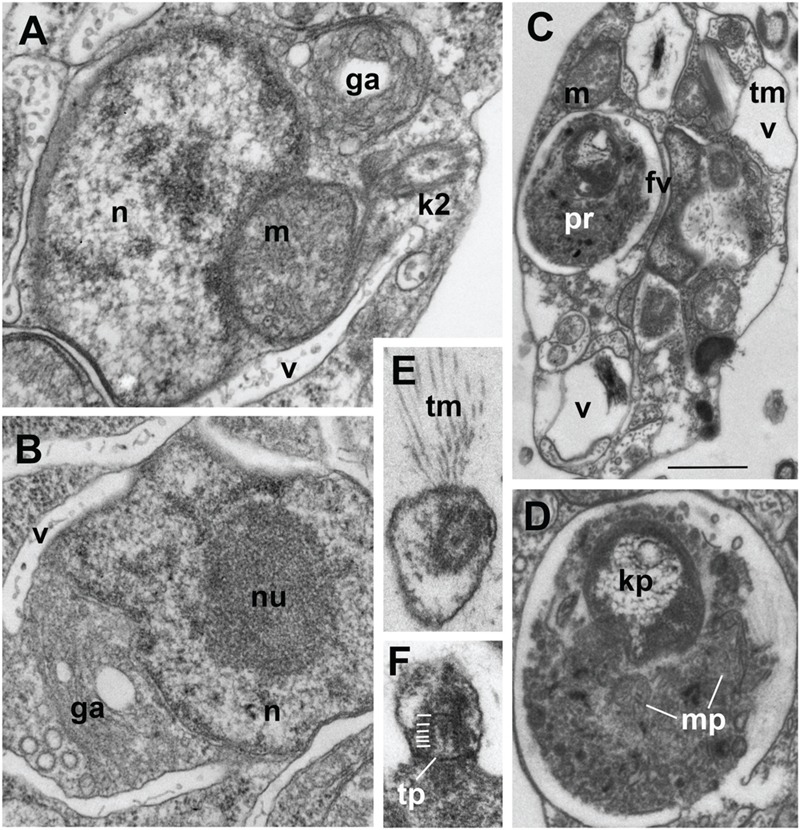
**Ultrastructure of *Develorapax marinus*. (A,B)** Nucleus and complex of associated organelles. **(C)** Food vacuole with partly digested kinetoplastid, **(D)** food vacuole at higher magnification with remained kinetoplast and mitochondrion, **(E)** unilateral tubular mastigonemes (tm), **(F)** tangential section of flagellar transition zone with six gyres of transitional helix. Scale bars: **(A,B)** 500 nm; **(C)** 1.5 μm; **(D–F)** 200 nm.

Unlike the bacterivorous *Developayella, D. marinus* is able to engulf free-living bodonids *Procryptobia sorokini*, which were used as a prey for the culture, with the posterior end of the cell. The cells of *D. marinus* often contain a huge food vacuole with remnants of the bodonid prey (**Figures 1C** and **2C,D**), and the cultures of *D. marinus* died off when the bodonid prey was depleted. The remnants of the bodonid mitochondrion with flat cristae and the kinetoplast were found in the food vacuole (**Figure 2D**). Most of the time the predator swims in a zig-zag motion, but sometimes makes broad circles hunting for prey. Sometimes the cells of *D. marinus* settle down to the bottom of the Petri dish staying immobile, but unlike *D. elegans* never attach to the substrate with a thread.

### Electron Microscopy

#### General View

**FIGURE 3:**
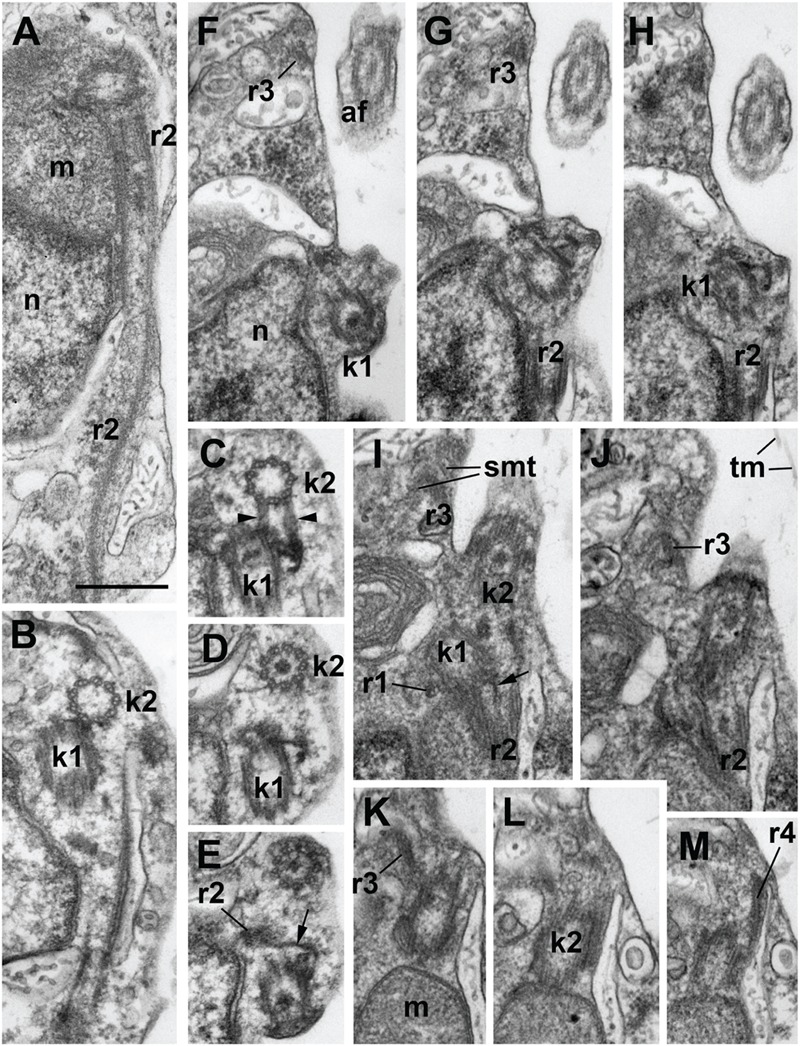
**Structure of kinetid in *Develorapax marinus*. (A)** Root 2 at longitudinal section. **(B–E)** Series of consecutive transverse sections of kinetosome 2. Arrowheads on **(C)** show two connectives between kinetosomes. **(F–M)** Series of consecutive longitudinal sections of kinetid. Arrows on **(E,F)** show the fibrillar strand of k1 becoming the core of r2. Scale bar: 400 nm.

Similar to the cells of *D. elegans*, the cells of *D. marinus* were not well preserved in whole mount preparations, e.g., the flagellar mastigonemes were not described by this method. But in the sections the typical heterokont tubular tripartite mastigonemes are present at one side of the anterior flagellum, i.e., anterior flagellum has unilateral mastigonemes (**Figures 2E** and **3I,J**). Vacuoles and dilations of endoplasmic reticulum with tubular mastigonemes are also commonly visible around the nucleus (**Figure 1F**).

The cell body of *D. marinus* is covered by the plasma membrane that is irregularly underlined by flattened broad vesicles similar to those observed in *D. elegans.* But unlike *D. elegans* these alveolar-like vesicles are not restricted to the anterior part of the cell and are also present in its posterior half (**Figures 1E** and **2C**). They are absent in some cytoplasmic regions, but there seems to be no rule for their position. Densely staining bodies are also present under the plasma membrane, but they are bigger, rarer, and have more irregular shape than those in *D. elegans*, and also occur toward the cell interior. These bodies are certainly membrane bounded, and are reminiscent of extrusomes when located near the plasma membrane (**Figures 1G,H**), however, we found no extruded bodies. We also did not find microtubules underlying the alveoli, which are observed in *D. elegans*, other than a few groups that seem to belong to the flagellar roots or their secondary microtubules.

#### Nucleus and Organelles

Structure and location of the nucleus, mitochondria and the Golgi apparatus are similar to those in *D. elegans.* Nucleus with prominent central nucleolus and developed heterochromatin is located slightly anterior to the body center (**Figures 1E** and **2A,B**). It is slightly elongated, but often has irregular shape on the sections. Mitochondria profiles with tubular cristae (some cristae contain axial fiber – **Figure 1F**) can be seen throughout the cell, but two of them are certainly associated with an anterior part of the nucleus: one is dorsal, another – more ventral, always associated with the kinetid (**Figures 1E,F** and **2A**). A Golgi dictyosome lies anterior to the nucleus, and close to the kinetid.

Numerous elongated and rounded vacuoles, the so called multivesicular bodies are located around the nucleus and in the posterior part of the cell, similar to *D. elegans* (Tong, 1995). Most of them do not contain any food objects, but many small vesicles with cytoplasm, or membranes can be present (**Figures 1E,G** and **2A–C**). Some vacuoles contain one or several bacteria (**Figure 2C**). Big vacuoles with prey at different stages of digestion also occur in the cell posterior where phagocytosis takes place (**Figures 1C** and **2C,D**). Large food vacuoles with eukaryotic prey occur much more often in *D. marinus* than small food vacuoles with bacteria. The efficiency of digestion of the ingested bacteria *Pseudomonas fluorescens*, which are not common to marine communities, remains unknown.

#### Kinetid Structure

The flagella and their kinetosomes are inserted at an angle of about 60° to each other in *D. elegans* and up to an angle of 120° in *D. marinus* (**Figures 3F–J**). In the latter the kinetosomes lay in different planes (**Figures 3B–D**) and are slightly apart from each other, while in *D. elegans* they lay in the same plane, and the proximal end of the posterior kinetosome is attached to the side of the anterior kinetosome. The kinetosomes in both species are connected to each other by several fibers (**Figures 3C,D,F–H**). They have the usual 9 × 3 structure with a cartwheel pattern at the base. The organelle position around the kinetosomes is characteristic and similar in both species: the kinetosome of the anterior flagellum (K2) terminates close to the edge of a Golgi body, attaching to the mitochondrion (**Figures 3K–M**), while the kinetosome of the posterior flagellum (K1) passes parallel to the mitochondrion, which lies against the ventral side of the anterior part of the nucleus (**Figure 3A**) (Tong, 1995).

*Develorapax marinus* has a flagellum with short transition zone containing a transitional plate at the cell surface level, and six gyres of a double transitional helix above the plate (**Figure 2F**). *D. elegans* has the same structure of flagellar transition zone, and the number of gyres is also 6 (Figure 9 in Tong, 1995).

A conspicuous microtubular root arranged in a semicircle around an electron-dense core was found in both species (**Figure 1E**). This root contains 10 microtubules and passes from kinetosome 1 posterior along the right margin of the ventral depression for about 1.8 μm in *D. elegans* (Tong, 1995). In *D. marinus* this root (R2) has eight microtubules. Other elements of the root system were not illustrated for *D. elegans*.

Kinetid structure was investigated for *D. marinus* on serial sections (**Figure 3**), showing organization of the roots. Kinetosome 1 has two microtubular roots: short inconspicuous r1 comprising 1–2 microtubules and passing almost perpendicular from K1 to the plasma membrane, and long and prominent R2 comprising eight microtubules and passing backward nearly parallel to the ventral cell surface. Root 2 forms a semicircle at cross sections and is associated with a fibrillar core, which lies nearly at the center of the semicircle. This core is, in fact, a distal end of the fiber originating from K1 (**Figures 3E,I**).

Kinetosome 2 also has two microtubular roots: r3, which comprises two microtubules, produces many secondary microtubules supporting the shape of the anterior end of the cell, and a rather short r4 also comprising 1–2 microtubules and passing to the cell anterior (**Figures 3J–M**).

Several fibrillar interkinetosomal connectives can be noticed (**Figures 3A,C–E,G,H**). Some of them have rather peculiar shape, other are long and underline the anterior/right depression of the cell, but they were not studied in details.

Kinetid structure of *Developayella* is similar to that of *Develorapax*. Having more information on *Develorapax* we can add that R2 has a reduced number of microtubules (8 vs. 10) and an angle between the kinetosomes is not stable: in trophic cells it is about 60°, but becomes wider during the transformation into the swimming cells where it can reach 120° (**Figures 3F–J**).

### Molecular Phylogeny

**FIGURE 4:**
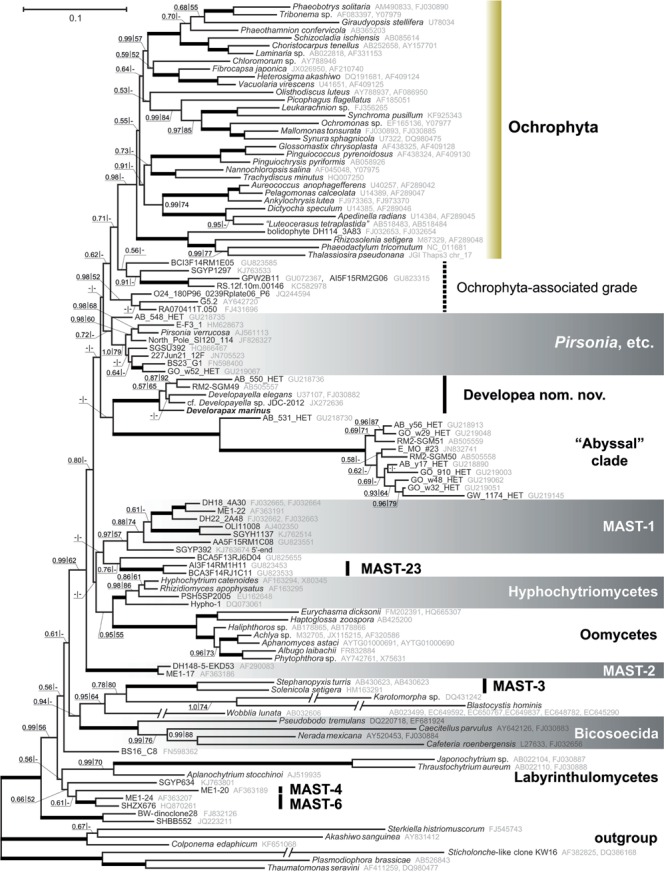
**Heterokont ribosomal RNA gene phylogeny with focus on the Gyrista clade.** Concatenated alignment of 18S and 28S rRNA genes with cumulative length of 4,606 positions was used for the analysis. The tree was reconstructed using the Bayesian Inference approach implemented by PhyloBayes under the CAT+GTR model with eight discrete gamma distributed rate categories. Nodes with Bayesian posterior probability of more than 0.9 and bootstrap support above 90% (RAxML) are marked with thicker lines. Resections mark branches shortened to half of their actual length.

The 18S and 28S rRNA gene sequences of *Develorapax marinus* and *Developayella elegans* have 94 and 91% identity within 1795 and 3280 overlapping sites, respectively. In Bayesian inference and ML trees the sequences are grouped with a *Developayella-*like nanoflagellate (del Campo et al., 2013) and a small cluster of environmental sequences (Scheckenbach et al., 2010; Takishita et al., 2010) with 1.0 posterior probability or 100% bootstrap support, respectively (**Figure 4** and Supplementary Figure S1). This group occupies an isolated position in the phylogenetic tree: in our analyses its closest relatives are a clade of derived environmental sample sequences from the abyssal plains of Atlantic and Pacific (Scheckenbach et al., 2010; Takishita et al., 2010; Jensen et al., 2012). The rDNA sequences of the latter clade are distinctly divergent and form a tight cluster with the exception of three environmental sequences (GU218726, GU218730, and GU2187370), which group with the “abyssal” clade in 90% of bootstrap replicates. These three almost identical sequences were generated in a single study and they passed a test for chimeric origin (Scheckenbach et al., 2010), but remain problematic in our analyses, as the inclusion of any of these three sequences in the dataset lowers the support for sister group relationship between the *Developayella*/*Develorapax* and the “abyssal” clades (Supplementary Figure S2). Ribosomal DNA sequences of the “abyssal” clade representatives are very divergent and can be a source of long branch attraction artifacts. Nevertheless, the removal of these sequences from the dataset has little effect in the overall topology of the tree (Supplementary Figure S3): the only noticeable difference in the tree topology is a switch in the relative positions of the *Developayella*/*Develorapax* and the *Pirsonia*-related branches.

More generally, the *Developayella*/*Develorapax* is a subclade within a much larger group of heterokonts – Gyrista *sensu* Cavalier-Smith and Scoble (2013). Omitting the unnamed clades of environmental sequences, the closest branch to ochrophytes in our analyses is *Pirsonia*, followed by the *Developayella*/*Develorapax* clade. Pseudofungi (oomycetes plus hyphochytriomycetes), MAST-1, and MAST-23 occupy an intermediate position between MAST-2 and the “crown” groups. The MAST-2 group is the first branch within the Gyrista in all analyses, although the support for the partition is only moderate. The support values for any of these partitions are low and insufficient for reaching confident conclusions about the exact branching order.

Another branch of colorless heterokonts, represented by environmental sample sequences and a well known parasitoid of diatom algae – *Pirsonia* (Schnepf and Schweikert, 1996; Schweikert and Schnepf, 1997; Kühn et al., 2004), diverges in between the *Developayella/Develorapax* cluster and the Ochrophyta (**Figure 4**). However, *Pirsonia*-related and *Developayella*/*Develorapax* branches are exchanged in the Bayesian tree when the taxon sample is changed (Supplementary Figure S3). Furthermore, these branches are sometimes grouped into one cluster with low support (not shown).

## Discussion

### Morphological Traits

*Develorapax marinus* differs from *Developayella elegans* by having larger cells, unilateral mastigonemes at the anterior flagellum, different kinetosome disposition: they lie in different planes, and have more varied angles; fewer microtubules (8 vs. 10) in R2; presence of dense bodies of irregular shape, and less ordered vacuoles. Cytoskeletal structures were not studied in detail in *D. elegans* therefore it is not possible to compare the R1, R3, and R4 roots.

The flagellar roots of *Develorapax* and obviously of *Developayella* are more similar to the ochrophyte flagellar root system, where each kinetosome has two microtubular roots. The most prominent long ventral root comprising 8–10 microtubules is, probably, homologous to the ventral root of *Wobblia* (or *Pendulomonas –* see Karpov et al., 2001) and *Placidia*, which are also arc-shaped in cross sections but composed of 10 microtubules with a core structure (Moriya et al., 2002).

One of the most conservative morphological characters for large scale phylogeny is the structure of the flagellar transition zone (Karpov and Fokin, 1995; Karpov, 2000). In this respect, both *Developayella* and *Develorapax* having the double helix are more similar to oomycetes than to the Ochrophyta and *Pirsonia* with an ordinary helix in the transition zone of flagella. A double helix was observed in *Placidia* flagellar transition zone (Moriya et al., 2002) therefore the state of this character in oomycetes, *Developayella*, and *Develorapax* is plesiomorphic relative to Ochrophyta and *Pirsonia*, and it is consistent with the position of these taxa in the phylogenetic tree (**Figure 4**).

The nearest described Ochrophyta neighbor is the *Pirsonia* – an amoeboid parasitoid of diatoms, which has biflagellate zoospores of heterokont type (Schnepf and Schweikert, 1996; Schweikert and Schnepf, 1997; Kühn et al., 2004). Zoospores of *Pirsonia* demonstrate features common to heterokonts: anterior flagellum with tripartite mastigonemes, tubular mitochondrial cristae, presence of an ordinary transitional helix in both flagella. At the same time their root system is unique: both microtubular roots of the anterior kinetosome (K2) are associated with fibrillar roots, and the posterior kinetosome (K1) produces only two fibrillar roots having no microtubular ones (Schnepf and Schweikert, 1996; Schweikert and Schnepf, 1997). Thus, the cytoskeleton of *Pirsonia’*s flagellates is rather unusual for heterokonts, and one can suggest, that this organism has reduced roots in specialized cells such as zoospores.

### Occurrence and Distribution

*Develorapax marinus* falls into a somewhat isolated heterokont cluster, along with the marine flagellate *Developayella elegans*. The flagellate *D. elegans* is a rare species: Among nearly 766 million raw rDNA sequence reads from 334 plankton samples collected during the circumglobal Tara Oceans expedition only 32 sequences from five samples have been found to belong to *D. elegans* or its close relatives (de Vargas et al., 2015). Nonetheless, *Developayella elegans* was registered after the first description (Tong, 1995) from marine sites worldwide (subtropical Australia, England and Korea [Lee, 2007]) at a wide range of depths (from surface water samples [Tong, 1997] to the depth of 467 m [Lee, 2007]) and salinity (from 28‰ [Tong, 1997] or freshwater [Lee and Patterson, 1998] to 67‰ [Patterson and Simpson, 1996; Al-Qassab et al., 2002]). *D. elegans* has been observed in planktonic and diverse benthic ecosystems (Lee and Patterson, 1998), in association with shallow microbial mats as well as with adjacent sediments (Al-Qassab et al., 2002). It can be hypothesized that a complex of cryptic highly specialized species is concealed under the name of “*D. elegans.*” Related to *D. elegans* sequences were found in plankton (de Vargas et al., 2015), microbial mats in deep-sea sulfide rich sediment associated with seeping fluids, Japan (Takishita et al., 2010), abyssal plains of the southeastern Atlantic (Scheckenbach et al., 2010), and from the Mediterranean plankton (del Campo et al., 2013). In the latter case, a stable culture of the bacteriotrophic *Developayella-*like nanoflagellates was isolated, and differences from the *D. elegans* in size and shape of the cells were detected, as well as differences in the 18S rRNA gene sequences (del Campo et al., 2013). *D. elegans* is the only described member of a whole group of morphologically diverged and ecologically specialized heterokonts.

### Endosymbiont Acquisition in Ochrophyta-Associated Heterotrophs

Rhodophyte-type plastids are found in many classes of Ochrophyta (Yoon et al., 2002), and the emergence of these plastids can be viewed as a relatively late event in heterokont evolution (Leipe et al., 1994, 1996; Stiller et al., 2014), as the majority of its early diverging lineages are heterotrophic, while autotrophs are located only within its diverse crown (Cavalier-Smith et al., 1994; Leipe et al., 1994, 1996; Guillou et al., 1999; Moriya et al., 2000, 2002; Karpov et al., 2001; Ben Ali et al., 2002; Marande et al., 2009; Riisberg et al., 2009; Yubuki et al., 2010, 2015; Harder et al., 2014). Eukaryotrophy has been documented for heterokonts outside Ochrophyta, the bicosoecids (*Cafeteria*), which can engulf a tiny *Ostreococcus*, which has the size of a bacterium (0.8 μm; Christaki et al., 2005). Unidentified thraustochytrids MAST-6 are likely able to ingest both algae (∼70%) and bacteria (up to 20%; Piwosz and Pernthaler, 2010). Other thraustochytrids Amphitremida seem to get the autotrophy independently the ochrophytes by the acquisition of green algal symbionts (Gomaa et al., 2013). All these groups are not close relatives to ochrophytes (**Figure 4**), and also can be considered as facultative predators, taking into account that the protists normally select the food particles by size and can eat both bacteria and picoeukaryotes. *Develorapax* is definitely an obligate predator engulfing large kinetoplastid cells by pseudopodia and its gene sequences branch off at the base of the Ochrophyta lineage. Thus, it can be suggested that it is a good candidate for a model of heterotrophic ancestor of the Ochrophyta.

The secondary loss of plastid hypothesis (Cavalier-Smith, 2002) is less parsimonious under the constraints of the topology obtained in our Bayesian tree, which also agrees with the trees obtained in previous studies. The list of heterotrophic lineages, in addition to *Pirsonia* (Kühn et al., 2004), MAST-1, MAST-2 (Massana et al., 2004, 2006, 2009), Pseudofungi, and *Developayella*, might also include a number of unnamed clades from the aphotic zones. The likely candidate is the “abyssal” clade, which is closely related to the *Developayella*/*Develorapax* group. Members of this clade were found in samples from white-colored microbial mats in deep-sea sulfide rich sediment associated with seeping fluids (Sagami Bay, Japan; Takishita et al., 2010), abyssal plains of the southeastern Atlantic (Scheckenbach et al., 2010), and in plankton from deep-water coral reef, Norwegian Sea (Jensen et al., 2012). Another candidate is MAST-23 (Massana et al., 2014), which are likely related to MAST-1 or Pseudofungi. Members of MAST-23 were found in a micro-oxic water column of the Caribbean Sea at depth of 200–900 m (Edgcomb et al., 2011). Micro-oxic habitats are not common for phototrophic eukaryotes, nor are deep water habitats, which hint at their likely heterotrophic lifestyle. The environmental sequences BCI3F14RM1E05, A95F15RM4A12, and their neighbors, which are grouped next to the ochrophytes (**Figure 4**), were also recovered from the same site in the Caribbean Sea. Another sequence related to the ochrophytes, GPW2B11, was found in an oxygen-depleted shallow sample from the Arabian Sea (Jebaraj et al., 2010). Two sequences with 100% identity (RA070411T.050 and RA070411T.002) were found in the total surface seawater sample, but were not detected in the sample after enrichment for photosynthetic organisms using flow cytometric cell sorting (Marie et al., 2010), therefore they are likely derived from non-photosynthetic organisms. The sequences of Ochrophyta-associated grade (**Figure 4**) are few in the environmental samples, and the conditions for the acquisition of some of them do not preclude their autotrophic lifestyle (Lefranc et al., 2005; Acosta et al., 2013; Lie et al., 2014) whereas for the other members of the grads are unambiguous indication of their heterotrophy. We suggest that bacterivory is the likely ancestral mode of phagocytosis as it is very common for the lineages of heterotrophic heterokonts. In this regard, *Develorapax* might be representative of the possible mechanism for endosymbiont acquisition in the ochrophyte lineage. We observed that the *D. marinus* tries to engulf the aggregates of bacterial cells in conditions of excessive bacterial mass in the culture medium, but it does not help to survive without eukaryotic prey. The formation of microcolonies or cell aggregates in plankton or on substrates is a common bacterial adaptive trait against protozoan grazing (Hahn et al., 2000; Jürgens and Matz, 2002; Matz et al., 2004; Matz and Kjelleberg, 2005; Jousset, 2012). However bodonids, which are common in eutrophic environments, and some other protozoans overcome this mechanism of bacterial protection by ingesting cell aggregates (Sibbald and Albright, 1988; Böhme et al., 2009; Erken et al., 2012). Members of the *Developayella/Develorapax* clade were found in the communities associated with bacterial mats (Al-Qassab et al., 2002; Takishita et al., 2010) or coral reefs (Jensen et al., 2012). It can be hypothesized that some bacterivorous members of eutrophic communities have acquired the ability to phagocytize not only individual bacterial cells but also their aggregates and larger eukaryotic prey. Ingestion of detritus is an option for food switching in predatory species (Pratt and Cairns, 1985), therefore bacterivory might reflect their ancestral trophic strategy. For example, MAST-6 strains are mixotrophic (algivorous and bacterivorous) in eutrophic water of the Southern Baltic Sea (Piwosz and Pernthaler, 2010) in contrast to the other known MAST organisms from oligotrophic waters that were shown to be bacterivorous (Massana et al., 2009). According to the rhodoplex (Petersen et al., 2014) or serial endosymbioses (Stiller et al., 2014) hypotheses, the Ochrophyta ancestor is suggested to be able to ingest an eukaryotic-sized cell of the cryptophyte-related endobiont precursor. Other scenarios, which suggest the origin of Ochrophyta by direct symbiosis with red algae, do not require a special adaptation to engulf eukaryotic cells because the cells of red algae can be very small. However, there are additional ways for prey to avoid the predators (Matz and Kjelleberg, 2005; Montagnes et al., 2008; Jousset, 2012). For example, the cells of the red microalgae are encapsulated within a cell wall composed of polysaccharides and glycoproteins, which protect the prey from recognition and digestion. This makes the cells of red algae unsuitable for a broad range of potential predators and may result in predator-prey specificity (Ucko et al., 1997, 1999; Martel, 2009). The scenario involving multiple symbiosis with rhodophytes requires independent symbiosis of one species of red algae with various hosts to explain the monophyletic plastid lineage (Yoon et al., 2002; Stiller et al., 2014) in the phyla Ochrophyta, Haptophyta, and Cryptophyta. Although the mechanisms for endosymbiont acquisition are far from being clear, the aforementioned adaptations of red algae against predation undermine the likelihood of direct symbioses with multiple distinct hosts. Unfortunately, the ability to engulf eukaryotic prey has not been tested experimentally for other members of the *Developayella/Develorapax* clade or any representatives of the Ochrophyta-associated grade. The *Developayella/Develorapax* clade is not sister to the ochrophytes in our trees and is unlikely to be representative of their immediate ancestor, but it may indicate the conditions under which an ancestor of ochrophytes acquired the ability to phagocytize eukaryotic prey and subsequently develop symbiotic relationship with a photosynthetic alga.

### Taxonomy

#### *Develorapax* Karpov et Aleoshin

Free swimming heterotrophic heterokont flagellate, predator. Anterior flagellum with unilateral mastigonemes, kinetosomes lie in different planes, with angles between kinetosomes from 60 to 120°. Root R2 comprises eight microtubules and forms an arc with a core at the proximal end at cross sections. Prominent flat or elongated vesicles with vermiform contents are abundant in cytoplasm; they do not form a layer of subplasmalemmal vesicles. The 18S and 28S sequences of *Develorapax marinus* have 94 and 91% identity, respectively, to those of *Developayella elegans*.

#### *D. marinus* Karpov et Aleoshin (**Figures 1–3**)

Cells are oval in shape, measuring 7–10 μm in length and 4–6 μm in width. Two flagella are inserted at the deepest part of a conspicuous depression at the right anterior half of the cell; anterior flagellum is 1.5 of the cell length, posterior flagellum is about twice the cell length; posterior flagellum is fastened along the bottom of the depression, while the anterior flagellum emerges freely. Flagellates are able to engulf free-living bodonids and do not produce a thread for attachment to the substrate.

Clonal culture Colp-4a was produced as a generation of a single cell isolated by a micropipette from a sample taken from the littoral of the Red Sea, 51°08′ northern latitude, 54°59′ eastern longitude, salinity 42‰ on the 4th of November, 2010. The culture is now extinct.

The blocks of epoxy resin with cells of *Develorapax marinus* are deposited at the culture collection of the Institute for the Biology of Inland Waters of the Russian Academy of Sciences (Borok, Yaroslavl district, Russia).

Earlier, Cavalier-Smith (1997) established class Bigyromonadea in phylum Bigyra to accommodate genus *Developayella*. The results of our analysis agree with a class level rank for the *Developayella*/*Develorapax* clade. However, the later division of the polyphyletic “Bigyra” assemblage *sensu* Cavalier-Smith (1997) lead to another, still polyphyletic, phylum “Bigyra” *sensu* Cavalier-Smith and Chao (2006), but now without the Bigyromonadea class. The International Code of Zoological Nomenclature does not enforce priority for higher level taxa, and to avoid possible confusion, caused by the association of the class name with the paraphyletic “Bigyra” nom. ambig., we propose a new class name for the *Developayella*/*Develorapax* clade – Developea nom. nov., including the order Developayellales and the family Developayellaceae (Cavalier-Smith, 1997).

The results of our analysis do not support the Developea+Oomycete (Leipe et al., 1996) or Developea+Pseudofungi group hypothesis (Cavalier-Smith and Chao, 2006). Furthermore, the extended sample of heterokont sequences studied here highlights the problems of reconstruction an accurate rDNA-based tree, and as yet insufficient sampling of the heterokont diversity, especially in close relatives of ochrophytes. At the same time, our results support the monophyly of a large group of heterokonts discovered earlier (Cavalier-Smith and Scoble, 2013), which includes Ochrophyta, *Pirsonia* and related environmental clones, Developea, Pseudofungi (oomycetes plus hyphochytriomycetes), MAST-1, MAST-23, another 3-4 isolated clades of environmental sample sequences, and MAST-2 as the first branch of the assemblage. Cavalier-Smith and Scoble (2013) referred to this large group as Gyrista, introduced earlier as a superphylum to accommodate Ochrophyta and “Bigyra,” and as a likely sister group to Sagenista, which includes Labyrinthulea and its closest relatives (Cavalier-Smith, 1997). The Sagenista were later included in the paraphyletic “Bigyra” (Cavalier-Smith and Chao, 2006; Cavalier-Smith and Scoble, 2013). In the ML tree from an earlier work (Cavalier-Smith and Scoble, 2013), Opalozoa, a part of the paraphyletic “Bigyra,” is sister to Gyrista. The same relationship can be observed in our Bayesian tree, albeit with poor support (**Figure 4**). Therefore, we consider it justified to keep the name Gyrista for the clade that is sister to Opalozoa, or alternatively recommend a new name, which would not be burdened by a history of previous revisions.

## Materials and Methods

Clonal culture of Colp-4a was produced as a generation of a single cell isolated by micropipette from the sample which has been taken from the littoral of the Red Sea, 51°08′ northern latitude, 54°59′ eastern longitude on 04th November 2010. The culture was maintained in Petri dishes with artificial marine medium Schmalz-Pratt (35‰; 28.15 g/l NaCl, 0.67 g/l KCl, 5.51 g/l MgCl_2_ × 6 H_2_O, 6.92 g/l MgSO_4_ × 7 H_2_O, 1.45 g/l CaCl_2_ × H_2_O, 0.1 g/l KNO_3_, and 0.01 g/l K_2_HPO_4_ × 3H_2_O in deionized water) without vitamins or microelements. The suspension of bacterivorous flagellates *Procryptobia sorokini* Frolov, Karpov et Mylnikov 2001 (Kinetoplastea) has been used as a food for Colp-4a. *P. sorokini* was fed by bacteria *Pseudomonas fluorescens* Migula.

Light microscopic (LM) observations were made with an AxioScope A1 (Carl Zeiss, Germany) using phase (PH) and differential interference contrast (DIC). The objectives of water immersion (40x and 70x) have been used. The microscope had analogous video camera AVT HORN MC-1009/S. The video frames have been directly digitized with tuner Behold TV 409 FM.

For transmission electron microscopy (TEM), cells were centrifuged and fixed in a cocktail of 0.6% glutaraldehyde and 2% OsO_4_ on Schmaltz-Pratt medium for 15–30 min at 1°C then dehydrated in alcohol and acetone series (30, 50, 70, 96, and 100%) for 10–30 min. Afterward, the cells were embedded in a mixture of Araldite and Epon (Spurr, 1969). Ultrathin sections were obtained with a LKB ultramicrotome and observed at JEM-1011 (Japan).

### DNA Extraction and Sequencing

The DNA was extracted with a Diatom DNA Prep kit (IsoGen Lab, Moscow). The rRNA gene fragments were amplified using Encyclo PCR kit (Evrogen, Moscow) and a set of primers (Medlin et al., 1988; Van der Auwera et al., 1994) using the following protocol: initial denaturation at 95°C for 3 min, 35 cycles of 92°C for 30 s, 55°C for 30 s, 72°C for 1.5 min, followed by a final extension period at 72°C for 10 min. PCR products were gel isolated and purified using the Cytokine DNA isolation kit (Cytokine, Russia) and cloned using the InsTAclone PCR Cloning Kit (Thermo Fisher Scientific). Clones containing rRNA gene fragments of *Develorapax marinus* and a prey *Procryptobia sorokini* were discriminated by sequencing. The *Develorapax marinus* rDNA sequences are deposited in GenBank under Accession Numbers KX500025–KX500026.

### Data Set Construction

Ribosomal DNA sequences of *Develorapax marinus* were aligned with 105 other members of Heterokonta found in the GenBank database, including uncultured clone sequences. Sequences were selected using the following scheme. First, we collected all heterokont large subunit rRNA genes outside of the Ochrophyta and included a few representatives from each class of the Ochrophyta, then we collected small subunit rRNA genes for all listed species if they were available. If the small subunit rRNA gene was missing for the selected species, a closely related species was used instead. Second, all fragments of heterokont small subunit rRNA genes outside of Labyrinthulomycetes that occupied isolated positions on the distance tree were selected from cultured strains and environmental samples. Third, two partially overlapping environmental sequences GPW2B11 (Accession Number GU072367) and A95F15RM4A12 (Accession Number GU823490) were concatenated into a single OTU because they both were found to be closely related to environmental sequence RS.12f.10m.00146 (Accession Number KC582978). Fourth, environmental sequence SGYP392 (Accession Number KJ763674), which is derived from a basal member of MAST-1 clade, was trimmed by 488 nucleotides from the 3′-end, which were derived from an ochrophyte species, a member of Dictyochophyceae: Florenciellales. Fifth, we removed the following sets of sequences: redundant sequences in the large clades, highly divergent sequences (*Rictus lutensis, Diplophrys* spp. etc.) branching far from the Developea, and environmental sequences, which were a suspect of chimeric origin (AY381178, GU219138, HQ867445, JN692703, JQ222877, KJ757848, KJ757946, KJ758005, KJ758134, KJ760760, KJ762852, KJ762875, KJ763015, KJ763027, KJ763520, and KJ763707). Three alveolate and three rhizarian species were chosen as an outgroup. Alignments were generated with MUSCLE (Edgar, 2004) and refined manually using BioEdit (Hall, 1999). After discarding ambiguously aligned nucleotide positions and concatenating the alignments of small and large subunit rRNA genes the alignment consisted of 4606 positions. GenBank Accession Numbers of the aligned sequences are given next to the sequence names in the tree (**Figure 4**).

### Phylogenetic Analysis

Tree search for the concatenated alignment was performed using the Bayesian method implemented by PhyloBayes version 3.2 (Lartillot et al., 2009) under the GTR+CAT+Γ8 model (Lartillot and Philippe, 2004) which was shown to be the least sensitive to systematic errors (Lartillot and Philippe, 2008). Four independent chains were run for 30,000 cycles sampling trees every 10 cycles and the first 2,000 points were discarded as burn-in. Bootstrap support values for the consensus tree reconstructed by PhyloBayes were generated using RAxML v. 7.2.6 (Stamatakis, 2006) on the basis of 1,000 replicates under the GTR+Γ+I model.

## Author Contributions

Conceived and designed the experiments: SK, AM, and VA. Performed the experiments: SK, AM, GM, KM, and VA. Analyzed the data: SK, AM, KM, VA. Writing of the manuscript: SK, AM, KM, and VA.

## Conflict of Interest Statement

The authors declare that the research was conducted in the absence of any commercial or financial relationships that could be construed as a potential conflict of interest.
