# Supplementary figures and images for: Heterokont Predator Develorapax marinus gen. et sp. nov. – A Model of the Ochrophyte Ancestor
Source: Front Microbiol. 2016 Aug 3;7:1194. doi: 10.3389/fmicb.2016.01194 (PMC4971089; doi:10.3389/fmicb.2016.01194)

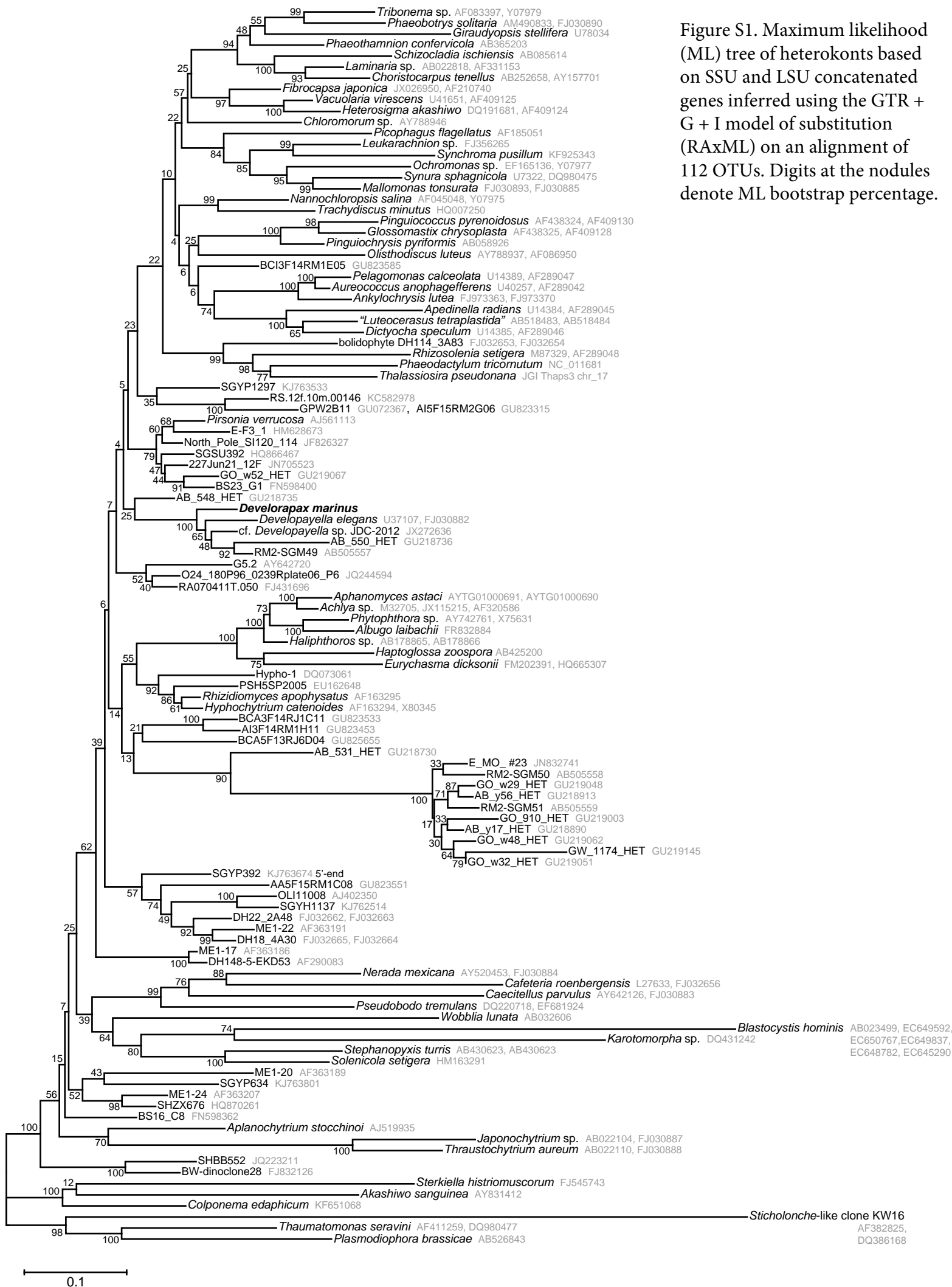

Supplement: Supplementary file 1 [file Image_1.PDF]

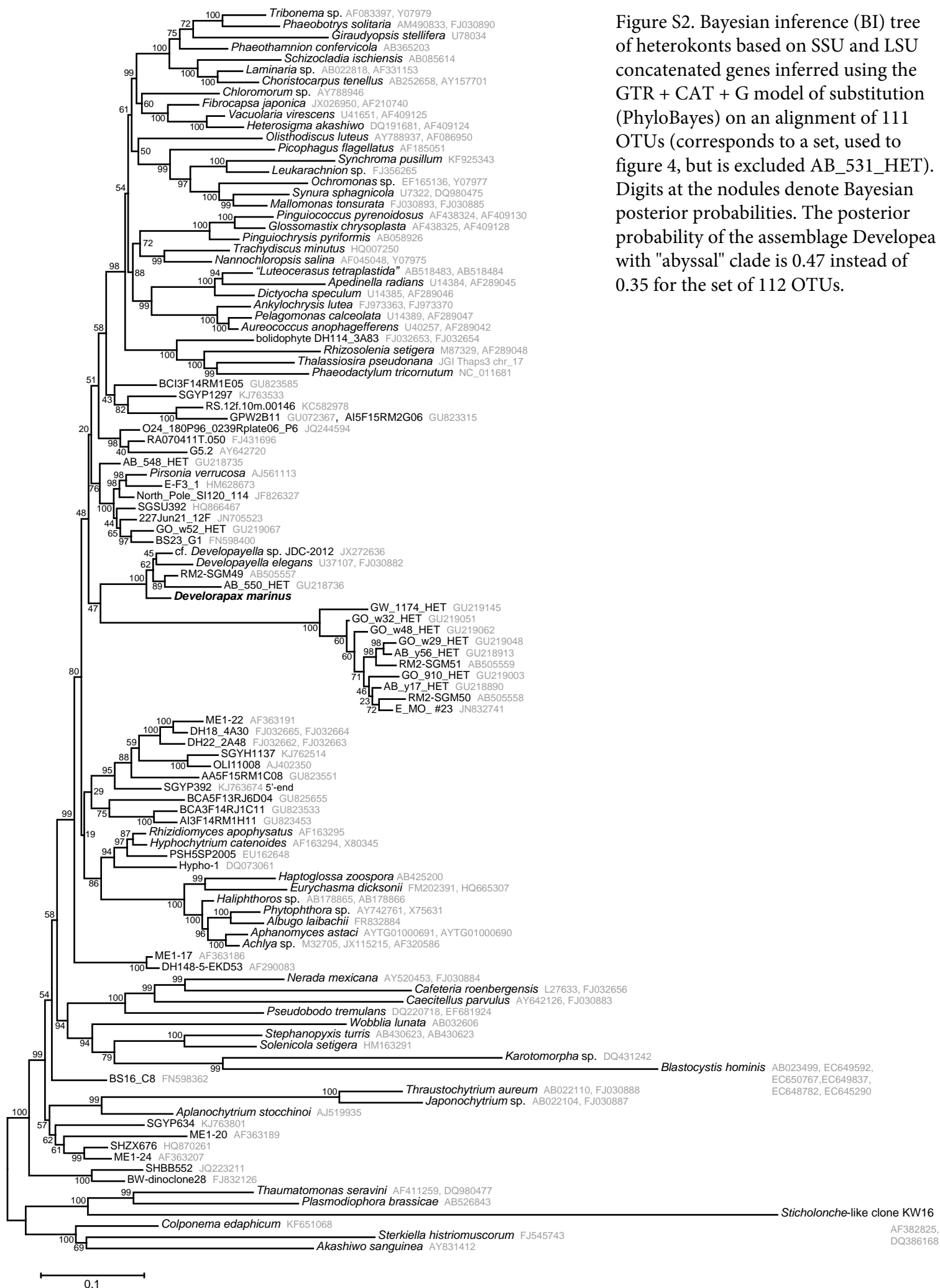

Supplement: Supplementary file 2 [file Image_2.PDF]

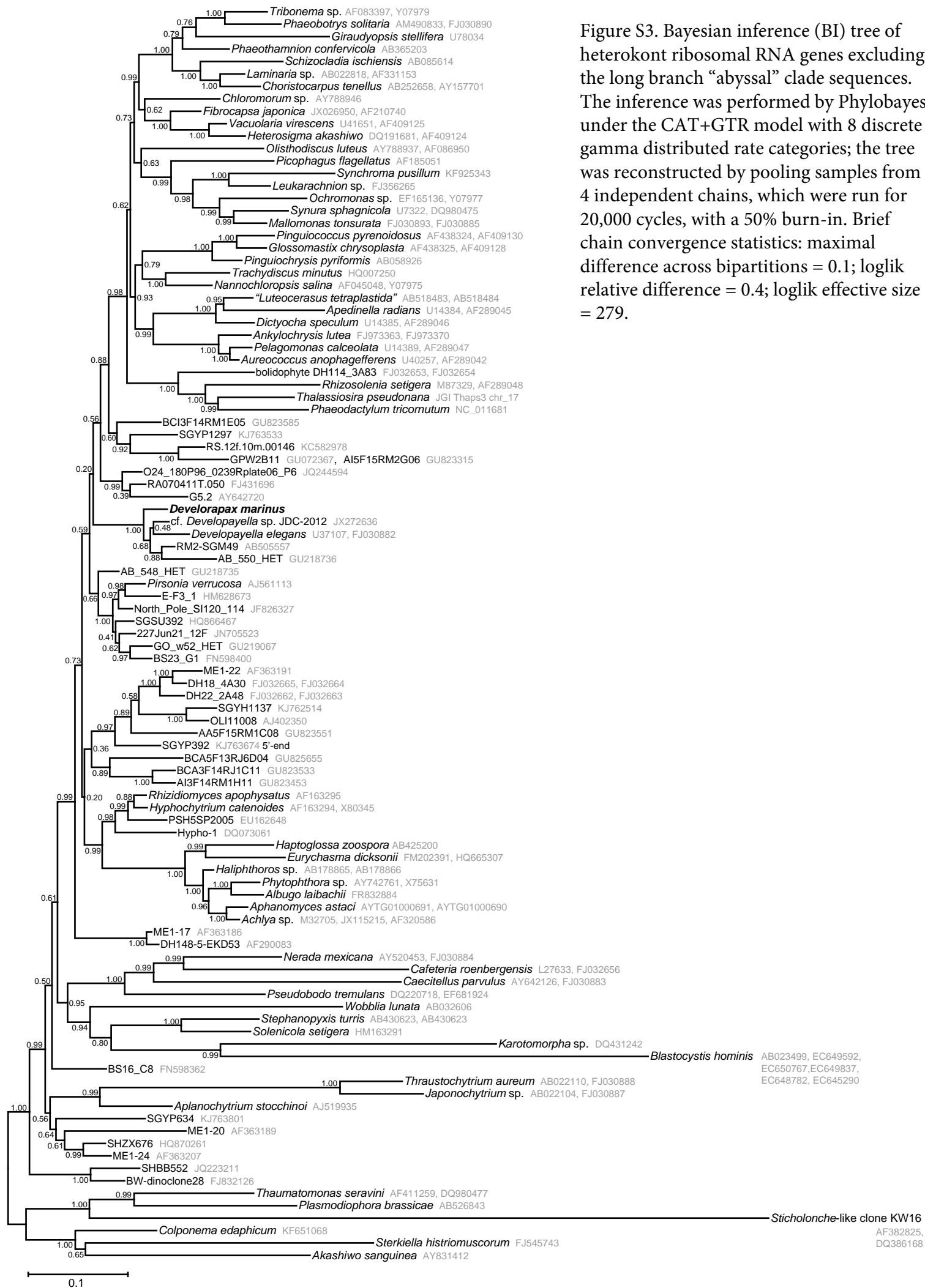

Supplement: Supplementary file 3 [file Image_3.PDF]
